# Factors affecting hand hygiene practice during the COVID-19 pandemic in the Zimbabwean population: a qualitative study

**DOI:** 10.1186/s12879-024-09277-1

**Published:** 2024-04-09

**Authors:** Nicholas Midzi, Masceline Jenipher Mutsaka-Makuvaza, Lincoln Sunganai Charimari, Priscilla Mangwiro, Tonderai Manengureni, Gladys Mugadza

**Affiliations:** 1 National Institute of Health Research, Ministry of Health and Child Care, Harare, Zimbabwe; 2 Department of Microbiology and Parasitology, School of Medicine and Pharmacy, University of Rwanda, Butare, Rwanda; 3 World Health Organization, Zimbabwe Country Office, Harare, Zimbabwe; 4 University of Zimbabwe, College of Health Sciences, Harare, Zimbabwe

**Keywords:** COVID-19, Hand hygiene, Hand washing, Facilitators, Barriers, Qualitative

## Abstract

**Background:**

Practicing hand hygiene is recommended as one of the key preventive measures for reducing the transmission of COVID-19 and other infectious agents. However, it is often not practiced frequently enough or correctly by the public. We aimed to identify barriers to and facilitators of hand hygiene in the Zimbabwean population during the COVID-19 pandemic.

**Methods:**

A qualitative study was conducted with a purposive sample of health workers, village health workers, church leaders, traditional healers, teachers, youth leaders and the general population selected from ten districts across the country from September to October 2022. Semistructured interviews were conducted with 3 key informant interviews per site. In addition, one homogenous focus group discussion was also conducted per site using a focus group discussion guide. The data were recorded on audiotapes, transcribed verbatim, and translated into English. All the analyses were performed manually using thematic analysis.

**Results:**

Two themes were identified as facilitators of hand hygiene. These include individual factors (knowledge of hand hygiene practices and how they are performed) and access-related factors (access to hand washing infrastructure, soap, and sanitizers). Among the barriers to hand hygiene, four themes were identified: individual factors (knowledge gaps in proper hand washing, lack of conviction about hand hygiene, and habitual behaviour), access-related factors (lack of access to hand washing infrastructure, soap, and sanitizers), safety concerns (concern about the side effects of sanitizers), and sociocultural and religious factors (social customs, cultural beliefs, values, and religious practices).

**Conclusion:**

During public health emergencies, there is a need for people to access uninterrupted, on-premises water supplies to promote compliance with hand hygiene. The provision of clean water and hand washing facilities is critical for vulnerable communities to afford them the opportunity to improve quality of life and facilitate resilience in the event of future pandemics. Community engagement is important for identifying vulnerability factors to provide appropriate mitigatory measures.

## Introduction

Coronavirus disease 2019 (COVID-19) caused by the SARS-CoV-2 virus was first detected in Wuhan city, China. It spread rapidly throughout China and across the world until it was declared a pandemic by the World Health Organization in March 2020 [1]. As of 30 June 2022, Zimbabwe had registered a cumulative number of 255 633 cases, 248 741 recoveries, & 5 557 deaths [2]. The SARS-CoV-2 virus spreads through direct means, such as droplet and human-to-human transmission, and by indirect contact, such as through contaminated objects and airborne contagion [3]. During the early stages of the pandemic, nonpharmaceutical interventions, such as the closure of various services and establishments, quarantine/isolation and restrictions on movement, and voluntary measures, supported by health promotion, such as disinfection of hands and surfaces, mask use, and maintaining a physical distance, were proposed to curb viral transmission [4]. In Zimbabwe, the Ministry of Health and Child Care guidelines for the management of COVID-19 specified the need for frequent hand washing with soap and water for at least 20 seconds or using an alcohol-based hand rub (60–80% alcohol content) among other COVID-19 prevention measures [5].

Hands play an important role in COVID-19 transmission as they come into direct contact with the mouth, nose and conjunctiva of the eyes, enabling the contraction of the virus [1, 6]. Thus, hand hygiene through sufficient hand washing with soap and water or hand sanitizing is recommended [6]. Hand washing has been implemented in most countries and is highly recommended for controlling infection and breaking the chain of COVID-19 transmission [7–9]. During the influenza pandemic, hand washing has also been proven to play a critical role in reducing disease transmission [10].

Adherence to hand washing depends on complex behavioural considerations, including social and cultural needs and environmental barriers [11–13]. A recent systematic review cited resource availability, cost and affordability, handwashing station design and infrastructure, accessibility, gender roles, health promotion, time management, knowledge, beliefs and behaviours as factors affecting hand washing in the community [14]. Low-income countries struggle to provide running water to many of their communities, thus compromising frequent hand washing. According to Brauer et al. [15], approximately 26% of the global population has inadequate hand washing facilities. The proportion of those in need of hand washing facilities is greater than 50% in Sub-Saharan Africa and Oceania. Approximately 64% of the Zimbabwean population has access to hand washing facilities on premises with soap and water services. The urban areas average 70% while the rural areas have approximately 60% [16, 17]. Disparities in handwashing have been observed between household heads with no education (47%) and those with higher education (82%) [16, 17].

Health workers in the country cited lack of running water in hospitals and communities for hand washing as one of the factors facilitating the spread of COVID-19 [18]. A lack of water was also cited as a barrier to hand washing in Chile [19]. In other settings, the presence of hand washing infrastructure in the household has been shown to be a determinant of hand washing practices [20]. In another study, people with access to hand washing facilities with soap and water were more likely to wash their hands with soap and water than those without access to these resources [21]. In Ghana, a lack of resources such as pipe-borne water and soap has resulted in people not being able to adhere to hand washing for the control of COVID-19 [22].

A community-based study conducted in 2020 in the Harare metropolitan province, Zimbabwe, documented positive attitudes and good practices toward hand washing in Harare in 2020 [23]. On the other hand, a prospective study conducted during the same year identified lack of hand hygiene facilities as one of the driving challenges of COVID-19 transmission in Zimbabwe [24]. However, the data on experienced facilitators of and barriers to hand washing in different settings in the Zimbabwean population are limited. This study aimed to provide an in-depth understanding of the facilitators of and barriers to handwashing among Zimbabwean communities during the COVID-19 pandemic. The lessons learned from this study will help in mitigating future pandemics.

## Methodology

### Study design and study setting

This was a descriptive phenomenology qualitative study. The study in Zimbabwe was conducted as a substudy of a larger study coordinated by the World Health Organization—Regional Office for Africa (WHO-AFRO), seeking to understand social-behavioural determinants of population compliance with public health and social measures and COVID-19 vaccine uptake in 6 selected African countries. Ten study districts were selected from 8 provinces based on vaccine uptake statistics in the District Health Information Software 2 (DHIS2) database. One of the objectives of the main study was to identify barriers to vaccine uptake. Thus, districts which had recorded lowest vaccine uptake in their respective provinces by the end of June 2022 were selected as study sites (Table 1). In the Harare metropolitan province, three districts were selected as study sites (Epworth, Mbare and Zengeza) because in addition to the province’s low vaccine uptake, compared to the other provinces, it had also recorded the highest number of COVID-19 cases (48 102) and deaths 1801) as of 30 June 2022 [2], (Table 1). The districts selected in Harare metropolitan province are known to have populations with diverse cultural backgrounds and tribes.

**Table 1:** COVID-19 magnitude in the provinces and vaccination coverage in study sites as of end of June 2022

**Province**	Number of COVID-19 cases (deaths)	Study d**istrict** /site	**Study group**	**COVID-19 vaccination coverage (%)**		
				**1st dose**	**2nd dose**	**3rd dose**
**Harare**	48 102 (1801)	Epworth	Women Leaders	47.1	35.2	5.5
**Harare**		Mbare	Transporters			
**Harare**		Chitungwiza -Zengeza	Religious Leaders	54.6	31.0	4.4
**Mashonaland Central**	14 409 (325)	Rushinga	Youth Leaders	43.1	27.1	1.0
**Masvingo**	21 915 (209	Chiredzi	Traditional Healers	32.0	30.9	4.5
**Mashonaland West**	31 376 (580)	Makonde	Village Health Workers	40.0	24.9	5.0
**Matabeleland North**	18 406 (131)	Binga	Health workers	35.7	35.7	4.3
**Matabeleland South**	17 886 (210)	Insiza	General Population	50.9	37.2	6.1
**Midlands**	18 147 (430)	Gokwe South	Teachers	42.5	22.3	3.7
**Mashonaland East**	33 482 (418)	Seke	General Population	36.7	27.8	3.7

Source of number of COVID-19 cases and death: [2]

The vaccine uptake of Epworth and Mbare district are combined in the DIHS2 database

**Fig. 1:**
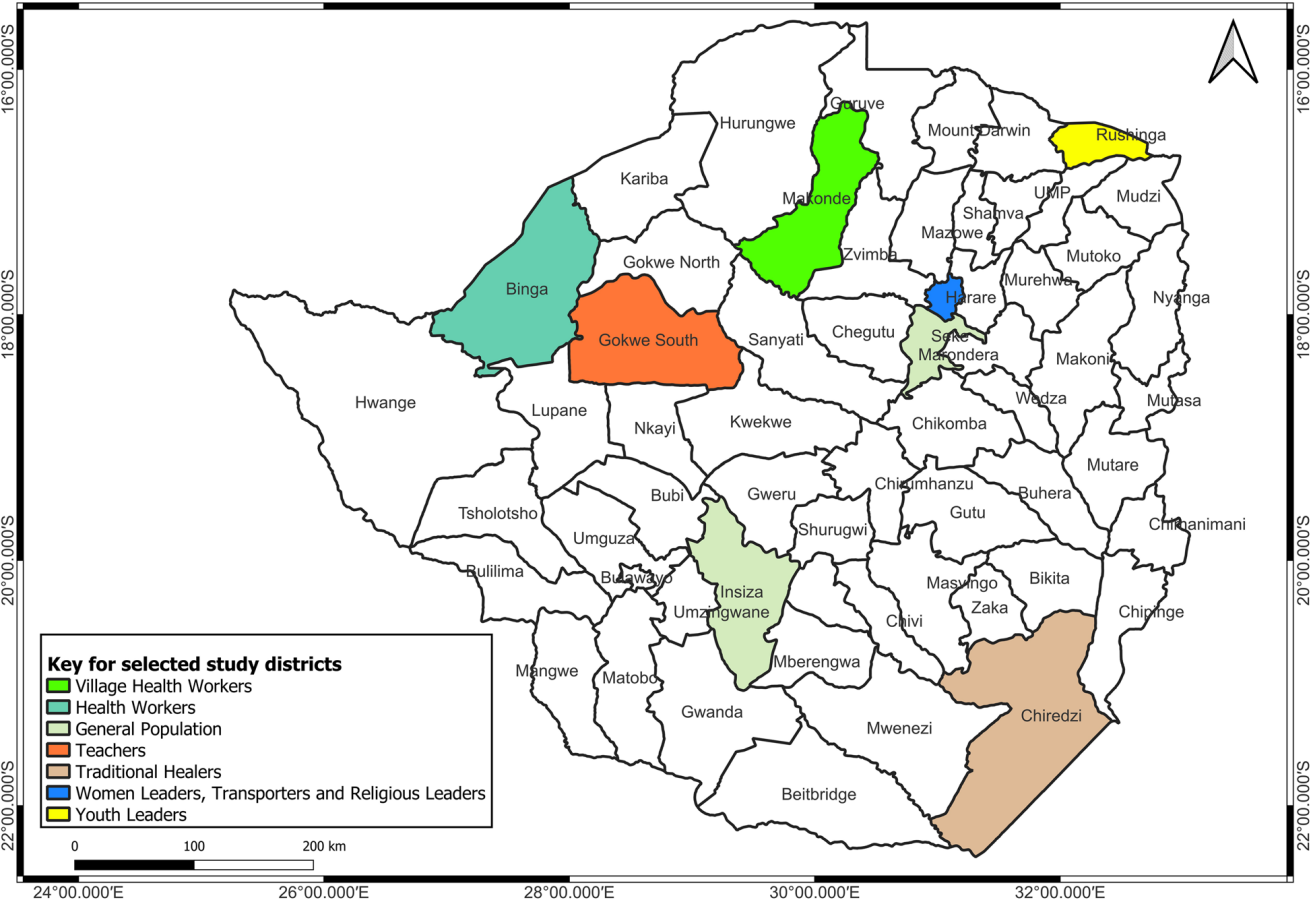
Location of the study sites and classification of study participants for the COVID-19 qualitative study in Zimbabwe

To capture a range of lived experiences and perspectives regarding COVID-19, the study included different social and economic population groups comprising health workers, village health workers, teachers, traditional healers, transporters, religious leaders, women leaders, youth leaders, and the general population (Table 1, Fig. 1). A homogenous group was selected per given study site to allow for data saturation with a sample of participants that is manageable and to enhance smooth coding and generation of themes.

The study groups were not replicated in all the study sites. The decision to sample specific groups in the specific areas was based on practical considerations including logistical feasibility, geographical and cultural diversity. For instance, Harare being a capital city, it is more populated and has many activities including women lead activities which can be conveniently found in vicinity and there is easy movement due to all time abundance of inexpensive transport, compared to the rural based study sites where people are widely spaced and their movement to the data collection points can be affected by transport logistics and bus fares. Thus, it was logistically sound and convenient to recruit women leaders in Harare to achieve the sample size than in other rural based provinces. Similarly, it was convenient to access religious leaders and transport operators in Harare because of the huge numbers of transport operators and existence of many church denominations in proximity in Harare allowing us to reach the sample size and minimizing dropouts based on distances to be travelled to the data collection point. Chiredzi district is known to have high numbers of traditional healers, thus it was convenient and feasible to recruit this group in this district. For the general population groups, we choose a peri-urban area (Seke district) and a rural area (Insiza district) to address power dynamics and inclusivity of marginalized populations, thus, capturing diverse perspectives and experiences. Village health workers were chosen to represent people’s perspectives and experiences in Makonde district since at one time Mashonaland West province, where the district is housed, experienced a huge surge in number of COVID-19 cases and village health workers had close interaction with the rural communities where they routinely provide health education for the control of COVID-19. Gokwe, Binga and Rushinga districts are some of the underrepresented communities in the country, thus choosing groups of people who hold influential positions such as teachers, health workers and youth leaders in these communities was considered that it would provide a broader reflection of community perspectives and experiences. Different study sites were used to allow triangulation as a strategy to enhance transferability of study findings.

In Harare metropolitan province, three study district; Mbare, Zengeza and Epworth, were chosen for the recruitment of transporters, religious leaders and women’s leaders, respectively. The three districts are high density, overpopulated areas housing residents of mixed culture and ethnic backgrounds. Epworth is a peri-urban district while Mbare district is located close to the central business district of Harare. Zengeza district is located in Chitungwiza, a dormitory town of the Harare metropolitan city. Vending and trading are the key activities in Mbare district. The district is also a central bus park station for transport connecting to different cities and districts and across the borders of Zimbabwe. Epworth district does not have proper ablution facilities. The three districts have limited hand washing facilities and the water supply is intermittent, resulting in people converging at central boreholes or tapes for water collection.

In Mashonaland West province, Makonde district was chosen as the study site. Village health workers were recruited for key informant interviews (KIIs) and focus group discussion (FGD) which were conducted at the growth point, which was previously a farming compound. Village health workers are community members trained and supported to provide basic health services and health education in their local communities or villages. A village health worker is part of the country's primary health care system, mainly in rural areas where access to health services is limited. There are no specific qualifications for village health workers but generally involve a combination of classroom training and on-the-job experience in community mobilization, health promotion, disease prevention, and basic first aid. They are generally expected to have qualities and attributes, such as good communication skills, empathy, and a commitment to serving their communities.

Functional tippy taps, referred to as *chigubhu gear* by the residents were observed at most households by the research team during key informant interviews and the focus group discussion.

In the Mashonaland East province, a peri-urban area situated close to Harare, Seke district was chosen as the study site for the general population study group.

Rushinga district, where youth leaders were chosen to participate in interviews and FGDs, is located in Mashonaland Central and borders Mozambique. Residents in the district have different cultural backgrounds. We observed that most of the shops at the growth point where the interviews and FGD were conducted had hand washing facilities.

The study district in Masvingo province, Chiredzi district, is inhabited by a population with different cultures and ethnic backgrounds. Data were collected in Chiredzi town with traditional healers as participants. The town which is densely populated, is central to the trade between Harare, Chiredzi, Masvingo town and South Africa. The town also experiences frequent water supply interruptions.

In Midlands province, Gokwe South district, school teachers were recruited for participation in the study. We observed that there was no hand washing facilities at the school entrances. We also noted that people, including school children, were not wearing face masks or observing physical distancing,

Binga district the study site in Matabeleland North province is a resort place attracting many tourists and fish traders. Most of the shops and buildings had hand washing facilities or sanitizers at their entrances.

In the Matabeleland South Province, the data were collected at the Insiza district growth point and at a rural area clinic in the same district. The rural clinic had a limited water supply, no convenient hand washing facility at the point of entry, and the Blair toilets were dirty.

### Sampling and sample size

Purposive sampling was employed at each site for the selection of participants who shared the same characteristics and had the potential to provide rich, relevant and diverse data pertinent to the research questions. For the 10 sites selected for the study, the targeted sample size was 30 key informants and 100 focus group discussion (FGD) participants. One FGD was conducted per site and homogeneity of the participants was considered to ensure data saturation. In total, 10 focus group discussions were conducted across the country. Each FGD comprised of a maximum of 10 participants. Generally, 4–12 participants are recommended per FGD [25]. Thus, the minimum number of participants expected per FGD was four. At each of the 10 sites, 3 key informants were purposively identified for the key informant interviews (KIIs). For each study group per site, the criteria for choosing key informants were: (i) having first-hand knowledge about their community, fellow residents, and issues or problems about COVID-19 based on their special social positions, experience, professional expertise, participation in a COVID-19 project or program previously conducted or currently running in the area (ii) being nonjudgmental and sensitive to differences within the community. Individuals fitting a study group per site were selected as FGD participants based on the criteria that they were able to express themselves in a group setting to provide a range of perspectives and experiences related to COVID-19 in the community. In addition for all participants, the feasibility of accessing and recruiting the participant, availability and willingness to sign the informed consent and participate in the study was also considered.

### Study guides and data collection

**S**tudy guides developed by the World Health Organization based on the literature on the uptake of vaccines were conceptualized for Zimbabwean settings by local researchers. Local researchers were composed of PhD experts in grounding theory studies and community health, individuals with master’s degrees in public health and research assistants with at least a degree in social sciences. Data collectors were trained by PhD experts in grounding theory studies and community health before the study was implemented. Key informant interviews and FGDs were conducted physically. Each FGD took approximately 45 min, and the KIIs were approximately 30 min each. The interviews and discussions were conducted either in English or in any of the two local languages, Shona or Ndebele. Each session was conducted by two moderators, and audio recordings were made for each session.

### Data analysis

All the audio recordings were transcribed verbatim. Local language recordings were translated into English after transcription. However some expressions were maintained in the local language. An English software could not be used since some of the local languages (Shona and Ndebele) expressions by participants which were critical as part of study findings would be lost as the software could not find any meaning out of them. The research team was familiar with the data to perform data coding and theme development manually. A thematic analysis approach was employed to develop an analytic template aligned with the FGD and KII guides as a preliminary point for analysis. An experienced qualitative researcher initially coded the transcripts, after which other investigators inputted into the codes while cross-checking the transcripts. The codes were initially clustered and subsequently sorted to create subthemes and, eventually, themes. The themes were captured and coded in Microsoft word, using a table, to show headings and bullet points which allowed for a visual representation of the data and facilitated the identification of patterns and trends. The manual coding facilitated a more-in-depth and nuanced analysis of the data identifying any discrepancies or inconsistencies in coding. The research team was able to engage in a thorough discussion of the themes and their association with the research question. To maintain rigour and validity, a reflexive approach was used to ensure consistency of the themes identified. Representative statements were drawn from the most important and repetitive quotes.

## Results

Of the expected sample size of 130 participants comprising 30 participants for KIIs and 100 participants for FGDs, 128 (98.4%) took part in the study. Two participants were lost to follow-up in 2 focus group discussions: one for traditional healers and the other for women leaders. All the other FGDs had 10 participants each.

Two themes were identified as facilitators of hand hygiene. These included individual factors (knowledge of hand hygiene practices and how they are performed) and access-related factors (access to hand washing infrastructure, soap, and sanitizers). Among the barriers, the four themes that were identified were individual factors (knowledge gaps in proper hand washing, lack of conviction about hand hygiene, and habitual behaviour), access-related factors (lack of access to hand washing infrastructure, soap, and sanitizers), safety concerns (concern about the side effects of sanitizers), and sociocultural and religious factors (social customs, cultural beliefs, values, and religious practices), all of which also contributed to poor compliance with hand hygiene among the Zimbabwean residents.

### Facilitators to compliance with hand washing

#### Knowledge/awareness

Awareness of proper hand washing and the use of alternative, affordable hand washing facilities have allowed the community to adhere to hand hygiene standards.

*“We were encouraged to do a proper hand wash with soap and water (participant demonstrating….). There was an introduction of the chigubhu giya (tippy tap), which aids hand washing without contaminating the water” (FGD participant 10, Makonde District, Village Health Worker).*

Different social and economic groups, such as religious leaders, traditional leaders and public transporters whose activities involved interacting with the general public, knew that they had to practice hand hygiene to prevent COVID-19 transmission.

*“––– about the shaking of hands, we are not to shake hands at all. –- Wherever one goes; to the supermarket or church, before entering the building, one should sanitize one’s hands. In fact, individuals are encouraged to use sanitizers for cleaning their hands at any time, if needed” (KI 3, Zengeza District, Religious Leader).*

*“They have to clean their hands with sanitizer before they can be assisted. –- After consulting, patients washed their hands with water and soap as they left. Handshaking was avoided as we greeted each other, observing the required physical distance.—When it came to assisting people in my surgery, I always wear hand gloves—even when we pray for our patients. Plastics, gloves – used were then disposed of after use” (KI 1, Chiredzi District, Traditional Healer).*

*“Hand washing with water and soap because hand get in contact with many surfaces, e.g., handling the steering wheel and all other places” (FGD participant 1, Mbare District, Transporter).*

#### Access to hand washing facilities

In other settings, it was mentioned that hand hygiene was not a problem since most facilities had hand washing equipment.

“We had no problem with hand hygiene. Most people had no problems washing their hands. They were now used to hand washing, as many shops and pharmacies had hand washing stations at each entrance to enable people to clean their hands to buy food and medication.” KI 1, Chiredzi District, Traditional Healer).

In rural areas where there were no taps, awareness of the importance of proper hand washing with running water was emphasized, resulting in the devising and use of homemade tippy taps. Villagers avoided contaminating surfaces or touching contaminated surfaces through the use of these tippy taps. They also resorted to storing small leftover soap bars for hand washing. Those who could afford bought hand sanitizers.

“The first is the one in the community that I live in, here in the rural areas. We encourage every household to have what we call chigubhu gear (tippy tap); it does not cost much. An old empty bottle of cooking oil can be used for hand washing as a chigubhu gear. This technology uses your foot to control it and let water out without anyone touching the same surface, hence preventing the spread of COVID-19. ––. We do not throw away these small leftover soaps; rather, we budget them for use in hand washing. –– Another measure for those who have the resources is that they buy sanitizers for themselves and their family members.––-moving around with a sanitizer for use even where there will be no access to hand washing facility and clean water” (KI 1, Makonde District, Female, Village Health Worker).

“Every homestead had a tippy tap for everyone entering the home to wash hands even at funerals there will be several tippy taps to ensure that people have access to washing hands” (FGD participant 5, Insiza District, Community Member).

In some localities, village health workers encourage residents to use facilities and items that are readily available for hand hygiene.

“Tools were available, though some would ask where they could get sanitizers, while others mentioned that they cannot afford the sanitizers. We would encourage the community to use water and soap, that we use at home or even use ash that is readily available at each and every homestead, so these are the measures that we advocated for and told the community not to worry about sanitizers that they could not afford but rather focus on things at their disposal” (KI 3, Makonde District, Village Health Worker).

### Barriers to hand hygiene

#### Knowledge gaps

Although some people practiced hand washing, they had no knowledge of the correct hand washing method.

*“Others were just used to ordinary hand washing and not following the scientific hand washing steps. Ordinary hand washing is most common, and people use it. People just continue rubbing their wet hands. It will take time for people to adopt the recommended hand washing steps” (FGD participant 2, Epiworth District, Women Leader).*

#### Habitual behaviour

In some instances, besides having available hand washing facilities, people would not wash their hands because they were not accustomed to frequent hand washing.

“–––-it was something that we were not used to. We were used to washing our hands when coming from home, and that was it. There were challenges in getting used to the new norm. Since it was something new, some people were not familiar with this practice, though others had adapted them” (KI 2, Rushinga District, Youth Leader).

“There was a challenge in observing the required frequency of washing hands. Mostly people would wash hands when they are eating only” (KI 1, Seke District, Development Economist)

#### Lack of conviction about hand hygiene

Despite the encouragement from village health workers to install hand washing facilities at all entrances of the homestead, for unknown reasons, some would not heed the call. In areas where hand washing facilities were available, some would just pass through without washing their hands.

“We would encourage that hand washing equipment (zvigubhu gear) be placed on all gathering entrances and homesteads, though some would not take head of the call. Even where hand washing points were available, some would just pass by without washing their hands for the sake of just being stubborn and not giving the measure the importance it deserves, while some would wash their hands” (KI 3, Makonde District, Village Health Worker).

The public transport workers indicated that although sanitizers were available, they would not use them and intentionally preferred to continue with their habits as before the COVID-19 era.

“At times, I will be away, and someone maintains the queue for me by moving the bus; they handle the steering with their hands, and that becomes a contaminated common surface. We had sanitizers, but we hardly used them” (FGD participant 6, Mbare District, Transporter).

#### Access challenges

Due to the scarcity of water in some localities, residents were unable to practice frequent hand washing. People had to travel long distances to fetch water; hence, they would try to limit its use. In areas where tap water was supposed to be available, it was intermittent due to interruptions in the electricity supply, resulting in people minimizing water use. Both rural and urban communities bemoaned water scarcity. It was also a problem in health care settings.

“The scarcity of sanitizers and inadequate water supply made hand washing and its frequency challenging. People in this locality fetch water from afar and may not afford to frequently perform a hand wash. The few people around who have tape water can manage to do frequent hand washing” (KI 2, Makonde District, Village Health Worker).

“On issues of hand washing, water and electricity are sometimes interrupted. In addition, we can go for a week or more without water. When power is shut down, water cuts follow because they use electricity as well. Therefore, even health workers cannot wash hands in the hospital, even when we talk about sanitation where everyone is carrying tissue anywhere just to relieve themselves. Therefore, the water situation even up to now hasn’t been resolved” (FGD respondent 4, Binga District, Health Worker).

“There was not enough necessary information needed. We have water shortages in our district just like in urban settings. People used their water sparingly, such that people would not regularly wash their hands.” (KI 1, Seke District, Development Economist).

Adherence to hand hygiene was also compromised by the nonavailability of soap and sanitizers. People try to make soapy water using small soap bars remaining after washing. Due to inaccessibility, some people have tried to produce homemade sanitizers whose formulation was not known to be effective.

“Hand hygiene faced challenges associated with nonavailability of soap and sanitizers. People tried to improvise making soapy water out of the tiny soap particles after the big soap bar has been used up. Getting powdered soap like surf was a challenge, and local people would at some point in time not afford a soap bar” (FGD respondent 9, Makonde district, Village Health worker).

“There was no recommended sanitizer formulation as they were homemade without supervision; thus, their effectiveness was questionable” (FGD participant 7, Seke District, Community Member).

Some traditional healers also echoed the limited water supply and lack of soap for their clients and themselves to practice hand washing.

“On that issue [hand washing], we had challenges. We usually have a single bucket and very little soap for hand washing, which would not be sufficient to cater to a large family” (KI 1, Chiredzi District, Traditional Healer).

Some public places had no hand washing facilities, making frequent hand washing at bus loading termini and other waiting areas a challenge. In some cases, hand washing facilities lacked water or soap.

“The challenge was on facilities. Additionally, some people forget to put in the water. When looking at it, we can say the problem was on facilities” (KI 2, Rushinga District, Youth Leader).

##### Concerns about the side effects of sanitizers

Some members of the community were reported to have experienced some side effects due to the use of sanitizers. Hand sanitizers had some side effects on some people, such as peeling of the skin, such that people decided not to use them.

*Sanitizers also affected some people through hand peeling. –––For sanitizers, some affected people’s hands by peeling their skin, possibly because of us trying to secure cheap products. As such, some people became disabled in the hand” (FGD participant 1, Gokwe South District, Teacher).*

#### Sociocultural factors and religious beliefs

Traditional healers mentioned that the ancestral spirits that possessed them were not aware of the requirements of hand washing with soap in line with COVID-19 preventive measures. This approach has allowed traditional healers to resort to alternative means of hand washing that were not proven to prevent COVID-19 transmission.

“Hand hygiene was a challenge, as the ancestral spirit does not know about hand washing. The other challenge was with the scent of modern soap, which was not compatible with the ancestral spirit, and we ended up using ruredzo (a local herb that mimics soap) to counter the challenge.––” (FGD participant 9, Chiredzi District, Traditional header).

Some church leaders indicated that hand hygiene was limiting their spiritual practices of hand laying, which resulted in compromised hand hygiene.

“—At times, depending upon the gravity of the situation, we would spray sanitizer to make the laying of hands less risky and at the same time enable prayer to be conducted in the normal fashion. The instruction from the Ministry of Health forbade us from the laying of hands during prayers, so this was a great impediment in our ministry at that point in time.” (KI 2, Zengeza District, Male, Pastor).

Culture and the value of maintaining close contacts and relationships were some of the factors that impose a challenge on maintaining social distance and hand hygiene. The communities were not able to practice these preventive measures at funerals, as people were accustomed to hand shaking or hugging as a way of consoling each other for losing a loved one. Even if hand washing facilities were available at some funerals, some people would just forgo hand washing.

“Hand hygiene was also a challenge—at funeral gatherings as people believe in hand shaking as an African cultural symbol for paying condolences. Without hand shaking as part of condolences, one would feel less comforted during the time of loss and bereavement. Amidst placement of water containers for hand washing, people would simply ignore the hand washing practice, and this was a challenge” (FGD respondent 2, Makonde District, Village Health Worker).

Based on cultural practices, it was also difficult to avoid hand shaking with the elderly people.

“In the areas where we stay, we also have the elderly and in laws who prefer handshakes as a way of greeting; therefore, we cannot avoid greeting them. Some even thought there was no COVID-19; hence, when you get to crowded places, especially funerals, there was no way you could avoid handshakes.” (FGD participant 3, Mbare district, transporter).

Due to sociocultural values, some people found it difficult to tell their visitors to wash their hands before they entered the house. Telling someone to wash hands was considered stigmatizing.

“It was difficult to return a visitor to go and wash their hands at the tippy tap, which is positioned at the entrance of the home because they would feel unwelcome; however, now, we can talk about washing hands without offending anyone as they understand the COVID-19 preventive measures. At first, telling someone to wash hands was like you are saying they brought COVID-19 to your home.” (FGD participant 5, Insiza District, Community Member).

## Discussion

This study explored contextual factors affecting hand hygiene during the COVID-19 pandemic in the Zimbabwean population using qualitative approaches to provide rich descriptions to be considered by public health managers as they design interventions for related epidemics. Facilitators of hand hygiene included individual factors such as knowledge of hand washing practices and accessibility of hand washing infrastructure, soap, and sanitizers. However, some members of the population were not able to adhere to hand hygiene practices due to knowledge gaps in proper hand washing, lack of conviction about hand hygiene, habitual behaviour, lack of access to hand washing infrastructure, soap and sanitizers. Additionally, concerns about the safety of hand sanitizers and sociocultural and religious factors leading to poor compliance with hand hygiene among the Zimbabwean residents were noted.

Knowledge and/or awareness of the importance of hand hygiene and how to perform hand washing were noted as internal motivators of hand hygiene among some people. On the other hand, a lack of knowledge of proper hand washing was noted as one of the internal barriers to hand hygiene. These findings corroborate previous reports in which knowledge was associated with hand hygiene practices [20, 26, 27]. A systematic review has also identified knowledge as one of the factors affecting hand washing in the community [14]. Moreover, having knowledge about COVID-19 has been shown to increase compliance with preventive measures, including hand washing [28]. Our findings also showed that some members of the communities lacked conviction on following hand hygiene practices and sometimes forgo hand washing or sanitizing even when resources are available. Such behaviour can lead to the transmission of COVID-19. Thus, to improve the knowledge and conviction of the community about hand washing and its importance, there is a need for improved provision of hand washing technique information to the community while also sensitizing them about the health consequences of a lack of hand hygiene. This will result in effective behaviour change. A rapid review has recently shown that risk communication can improve cognitive and behaviour change around COVID-19 [29].

Some people in the communities had certain habitual behaviours, resulting in them forgetting to frequently wash their hands. Other studies have also shown that participants forget to hand wash frequently [27, 30]. In such settings, hand washing awareness should be coupled with providing reminders to communities on frequent hand washing. Such reminders can be provided via text messages, roadshows or other avenues of communication suitable for the community.

The environmental determinants of hand hygiene practices, such as the availability and accessibility of hand washing infrastructure, soaps and sanitizers, were identified as some of the factors related to hand washing among the communities. It has been noted that some community members did not have money to buy soaps or sanitizers, while in some public places, hand hygiene facilities were not available. Those with these resources mentioned that hand hygiene was not a challenge, while the absence of these hand washing facilities, soap and/or sanitizer were cited as barriers to hand hygiene. These environmental determinants have also been noted as barriers to hand washing in Ghana among rural and urban slum dwellers [22, 31], among slum dwellers in Kenya [32], young adult students in Canada [27] and in poor settlements in Accra and Johannesburg [33]. A previous study in Zimbabwe also identified a lack of hand hygiene facilities as one of the challenges driving SARS-CoV-2 transmission in the country [24]. Like what has been noted in this study, a study among health workers in Zimbabwe identified lack of running water in the community in health care settings as one of the factors fuelling transmission of COVID-19 in the country [18]. Intermittent and insufficient water for hand washing during the COVID-19 pandemic was also reported in India [34]. Thus, vulnerable communities are sometimes willing to comply with hand hygiene and other preventive measures but lack the resources to do so. Thus, knowledge training should be accompanied by the provision of resources to ensure compliance.

As a coping mechanism, rural communities adopted locally appropriate hand washing aids, thus facilitating compliance with hand washing. As was indicated in some settings in this study, the household was supposed to have at least one tippy tap. A similar approach has also been noted in Ghana [22, 31], and these tippy taps have been noted to improve hand washing. While this mitigatory measure is welcome, such vulnerable communities should be considered for the provision of long-term mitigatory measures, as the tippy tap does not carry much water and thus would require frequent refilling.

The study findings show that community practices and values influence the observance of hand washing. Cultural values of promoting togetherness, close interaction, bonding, and belief in respecting ancestral spirits and hand laying as a spiritual healing measure contributed to poor observance of hand hygiene. Hand shaking at funerals is a cultural way of consoling the loved ones of the deceased. In Ghana [22], Pakistan [35], and Iran [36, 37], it was also reported that community members lifted COVID-19 restrictions, favouring continuing with sociocultural factors such as attending funerals, churches and family functions. Thus, it is important to engage the community when engaging in public health interventions so that people are aware that their cultural, religious or social diversity is taken into consideration in intervention measures.

Findings from our study showed that some people experienced skin peeling due to the use of sanitizers. While the communities suspected that this could be due to improperly constituted sanitizers, the reported concern about the safety of sanitizers cannot be disputed considering the documented evidence of their various side effects [38]. It has been documented that frequent and prolonged use of ethanol-based hand sanitizers may lead to health hazards such as irritation and allergic conditions of the skin and eyes and dryness or cracking of the skin with peeling, redness or itching [38, 39]. Thus, if communities witness or experience any side effects due to sanitizer use, they may forgo hand hygiene, especially in the absence of other hand washing resources such as water and soap.

### Study strengths and limitations

Triangulation through the use of KIIs and FGDs and different data sources (diversity in age, religion, social status, cultural values and economic status) from different sites in different settings (urban, peri-urban, growth point, rural and farming area) increased the trustworthiness and validity of the study, thus providing a comprehensive understanding of facilitators of and barriers to the observance of hand hygiene during the COVID-19 pandemic. Nonetheless, the study was limited in that it was cross-sectional; thus, it may not truly reflect the dynamic nature of social behavioural factors affecting hand hygiene in the Zimbabwean population.

## Conclusion and recommendations

There is need for access to an uninterrupted, on-premises water supply during public health emergencies to increase compliance with hand hygiene among people. Alongside other community development projects, the provision of clean water and hand washing facilities is critical for vulnerable communities to afford them the opportunity to improve quality of life and facilitate resilience in the event of future pandemics. To identify vulnerability factors that are likely to be faced with communities during public health emergencies, the government should engage communities so that they can provide appropriate mitigatory measures. The lessons learned during the COVID-19 pandemic should be used to intensify long-term community development.

## Data Availability

For the sake of protecting participant confidentiality, the audio recordings generated in this study are not available to the public. Anonymized transcripts are available from the corresponding author upon reasonable request.

## Abbreviations

COVID-19: Coronavirus disease 2019
DHIS2: District Health Information Software 2
FGD: Focus group discussion
KII: Key informant interview

## References

[1] Cucinotta D, Vanelli M (2020). WHO Declares COVID-19 a Pandemic. Acta Biomedica Atenei Parmensis.

[2] Zimbabwe COVID-19 Situation Report. 2022. 10.23750/abm.v91i1.9397.

[3] Lotfi M, Hamblin MR, Rezaei N (2020). COVID-19: Transmission, prevention, and potential therapeutic opportunities. Clin Chim Acta..

[4] Seale H, Dyer CEF, Abdi I. et al. Improving the impact of nonpharmaceutical interventions during COVID-19: examining the factors that influence engagement and the impact on individuals. BMC Infect Dis. 2020;20(607). 10.1186/s12879-020-05340-9.10.1186/s12879-020-05340-9 PMC7430133 32807087

[5] Zimbabwe guidelines for management of COVID-19. 2020.

[6] Haston JC, Miller GF, Berendes DM, Andújar AA, Marshall B, Cope JR, Hunter CM, Robinson BM, Hill VR, Garcia-Williams AG (2020). Characteristics Associated with Adults Remembering to Wash Hands in Multiple Situations Before and During the COVID-19 Pandemic — United States, October 2019 and June 2020. Morb Mortal Wkly Rep.

[7] Adola SG, Degavi G, Edwin SEK, Utura T, Gemede U, Kasimayan P (2021). Assessment of factors affecting practice towards COVID-19 among health care workers in health care facility of West Guji Zone, South Ethiopia, 2020. Pan Afr Med J.

[8] Beale S, Johnson AM, Zambon M, Hayward AC, Fragaszy EB, Flu Watch Group (2021). Hand Hygiene Practices and the Risk of Human Coronavirus Infections in a UK Community Cohort. Wellcome Open Res.

[9] Siswanto A, Murti B, Prasetya H. Face Mask Wearing and Hand Washing Behavior on the Prevention of COVID-19 Infection: A Meta-Analysis. J Health Prom Behav. 2022;7:182–96.

[10] Saunders-Hastings P, Crispo JA, Sikora L, Krewski D (2017). Effectiveness of personal protective measures in reducing pandemic influenza transmission: A systematic review and meta-analysis. Epidemics.

[11] Drankiewicz D, Dundes L (2003). Handwashing among female college students. Am J Infect Control.

[12] Jumaa PA (2005). Hand hygiene: simple and complex. Int J Infect Dis.

[13] Scott B, Curtis V, Rabie T, Garbrah-Aidoo N (2007). Health in our hands, but not in our heads: understanding hygiene motivation in Ghana. Health Policy Plan.

[14] Ezezika O, Heng J, Fatima K, Mohamed A, Barrett K (2023). What are the barriers and facilitators to community handwashing with water and soap? A systematic review. PLOS Global Public Health.

[15] Brauer M, Zhao JT, Bennitt FB, Stanaway JD. Global Access to Handwashing: Implications for COVID-19 Control in Low-Income Countries. Environ Health Perspect. 2020;128:057005. 10.1289/EHP7200 PMC7263456 32438824

[16] Government of Zimbabwe. Water, Sanitation and Hygiene (WASH) Sector 2019 Joint Sector Review. Theme: Sustainable WASH Services for Zimbabwe’s Economic Recovery Towards the SDGs. 17 – 18 October 2019, Mutare. 2019.

[17] UNICEF (2019). The State of Wash Financing in Eastern and Southern Africa: Zimbabwe Country Level Assessment.

[18] Mathew N, Rumbidzai C, Fungisai M. Exploring factors enabling the spread of COVID‐19: Narratives of health professionals in Harare, Zimbabwe. Health Soc Care Commun. 2022;30:e2973–9. 10.1111/hsc.13742 PMC9111785 35133044

[19] Obach A, Cabieses B, Vezzani F (2023). Perceived barriers and facilitators for adhering to COVID-19 preventive measures in Chile: a qualitative study in three large cities. BMC Infect Dis.

[20] White S, Thorseth AH, Dreibelbis R, Curtis V (2020). The determinants of handwashing behaviour in domestic settings: An integrative systematic review. Int J Hyg Environ Health.

[21] Wolf J, Johnston R, Freeman MC, Ram PK, Slaymaker T, Laurenz E, Prüss-Ustün A (2019). Handwashing with soap after potential faecal contact: global, regional and country estimates. Int J Epidemiol.

[22] Aberese-Ako M, Immurana M, Dalaba MA, Anumu FEY, Ofosu A, Gyapong M (2023). An Ethnographic Study of Multiple Factors Influencing Perceptions, Attitudes, and Observance of COVID-19 Preventive Measures among Rural and Urban Slum Dwellers in Ghana. J Environ Public Health.

[23] Gadzayi MR, Mubonani G, Muza A, Muwishi C, Chirongoma F, Nyangani N, Majuru E, Dube F, Pomo S, Dhlandhlara B, Muparanyama L, Govha E, Gombe NT, Juru T, Tshimanga M (2023). Knowledge, Attitudes and Practices on COVID-19 among urban residents, Harare, Zimbabwe 2020: A Cross Sectional Study. J Int Epidemiol Public Health..

[24] Murewanhema G. COVID-19 control pitfalls and challenges and drivers of SARS-CoV-2 transmission in Zimbabwe. Pan Afr Med J. 2021:38(28). 10.11604/pamj.2021.38.28.25758. 10.11604/pamj.2021.38.28.25758 PMC7955594 33777296

[25] Tong A, Sainsbury P, Craig J (2007). Consolidated criteria for reporting qualitative research (COREQ): a 32-item checklist for interviews and focus groups. Int J Qual Health Care.

[26] Tao SY, Cheng YL, Lu Y, Hu YH, Chen DF (2013). Handwashing behaviour among Chinese adults: a cross-sectional study in five provinces. Public Health.

[27] Thaivalappil A, Young I, Pearl DL, McWhirter JE, Papadopoulos A. “I Can Sense When My Hands Need Washing”: A Qualitative Study and Thematic Analysis of Factors Affecting Young Adults’ Hand Hygiene. Environ Health Insights. 2022;16. 10.1177/11786302221129955. 10.1177/11786302221129955 PMC9575434 36262200

[28] Soltan EM, El-Zoghby SM, Salama HM (2020). Knowledge, Risk Perception, and Preventive Behaviors Related to COVID-19 Pandemic Among Undergraduate Medical Students in Egypt. SN Compr Clin Med.

[29] Winograd DM, Fresquez CL, Egli M, Peterson EK, Lombardi AR, Megale A, Tineo YAC, Verile MG, Phillips AL, Breland JY, Santos S, McAndrew LM (2021). Rapid review of virus risk communication interventions: Directions for COVID-19. Patient Educ Couns.

[30] Scott E, Vanick K (2007). A survey of hand hygiene practices on a residential college campus. Am J Infect Control.

[31] Aberese-Ako M, Immurana M, Dalaba MA, Anumu FEY, Ofosu A, Gyapong M (2022). The socioeconomic and health effects of COVID-19 among rural and urban-slum dwellers in Ghana: A mixed methods approach. PLoS ONE.

[32] Oluoch K, Muthuka JK, Wabmura F, Muthoka M, Dede E, Ndukanio S. Challenges Of Adhering To Hand Washing Protocols As A Covid-19 Prevention Measure Among Slum Dwellers In Nairobi, Kenya. Eur J Public Health Stud. 2023;38:31–40.

[33] Durizzo K, Asiedu E, Van der Merwe A, Van Niekerk A, Günther I (2021). Managing the COVID-19 pandemic in poor urban neighborhoods: The case of Accra and Johannesburg. World Dev.

[34] Kumpel E, Billava N, Nayak N, Ercumen A (2021). Water use behaviors and water access in intermittent and continuous water supply areas during the COVID-19 pandemic. J Water Health.

[35] Ali I, Saddique S, Ali S. Local Perceptions of COVID-19 in Pakistan's Sindh Province: "Political Game", Supernatural Test, or Western Conspiracy? Disaster Med Public Health Preparedness. 2021;1–6. Advance online publication. 10.1017/dmp.2021.220. 10.1017/dmp.2021.220 PMC8438506 34247693

[36] SoleimanvandiAzar N, Irandoost SF, Ahmadi S, Xosravi T, Ranjbar H, Mansourian M, Yoosefi Lebni J (2021). Explaining the reasons for not maintaining the health guidelines to prevent COVID-19 in high-risk jobs: a qualitative study in Iran. BMC Public Health.

[37] Ahmadi S, Jorjoran Shushtari Z, Shirazikhah M, Biglarian A, Irandoost SF, Paykani T, Almasi A, Rajabi-Gilan N, Mehedi N, Salimi Y (2022). Social Determinants of Adherence to COVID-19 Preventive Guidelines in Iran: A Qualitative Study. Inquiry.

[38] Mahmood A, Eqan M, Pervez S, Alghamdi HA, Tabinda AB, Yasar A, Brindhadevi K, Pugazhendhi A (2020). COVID-19 and frequent use of hand sanitizers; human health and environmental hazards by exposure pathways. Sci Total Environ.

[39] Lachenmeier DW (2008). Safety evaluation of topical applications of ethanol on the skin and inside the oral cavity. J Occup Med Toxicol (London, England).

